# Severe psoriasis relapse following tralokinumab therapy for atopic dermatitis

**DOI:** 10.1016/j.jdcr.2026.05.062

**Published:** 2026-06-03

**Authors:** Nikkia Zarabian, Mina Farah, Joseph Zahn, Adam Friedman

**Affiliations:** The George Washington University School of Medicine and Health Sciences, Department of Dermatology, Washington, DC

**Keywords:** Atopic Dermatitis, Psoriasis, Tralokinumab

## Introduction

Atopic dermatitis (AD) is a chronic inflammatory dermatosis that affects nearly 18 million adults in the United States.^1^ Advanced understanding of the complex pathophysiology of AD has led to the development of targeted biologic therapies for the management of moderate-to-severe AD.^2^ Tralokinumab, a biologic agent currently approved by the U.S. Food and Drug Administration (FDA) for the treatment of patients 12 years and older with moderate to severe AD, selectively targets interleukin (IL)-13 by inhibiting its interaction with the IL-13Rα1/IL-4Rα receptor complex.^3^ Clinical trials have demonstrated a favorable safety profile for tralokinumab, with the most commonly reported adverse events including upper respiratory tract infections, conjunctivitis, and injection-site reactions.^3^ In this case report, we present a rare and poorly understood adverse event associated with tralokinumab, highlighting the diagnostic evaluation and management of a patient who developed a severe relapse of psoriasis following treatment for AD.

## Case report

**Fig 1:**
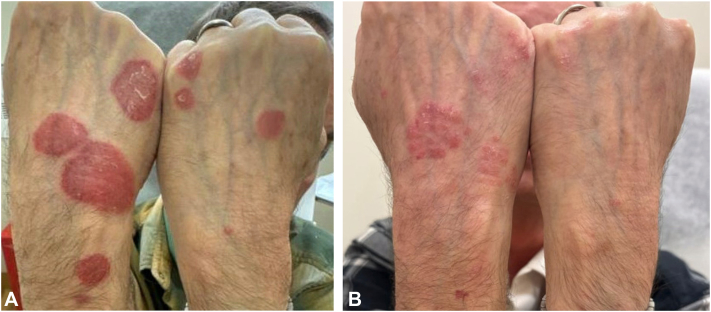
Clinical presentation of psoriasis at **(A)** initial presentation and **(B)** 16 weeks after upadacitinib initiation.

A 64-year-old male with adult-onset AD, characterized by chronic pruritus and ill-defined, scaly, erythematous plaques involving the trunk and upper/lower extremities, was treated with topical corticosteroids and dupilumab 300 mg subcutaneous injection every 2 weeks, with an insufficient therapeutic response. He was subsequently transitioned to tralokinumab 300 mg subcutaneous injections every 2 weeks, which resulted in complete disease clearance for 24 months. It was at this point the patient presented to clinic with pruritic, well-demarcated, erythematous scaly plaques distributed primarily across the lower extremities, elbows, dorsal hands, and ventral forearms (Body Surface Area (BSA) 15%, Physician Global Assessment (PGA) 4) (Fig 1, *A*).

**Fig 2:**
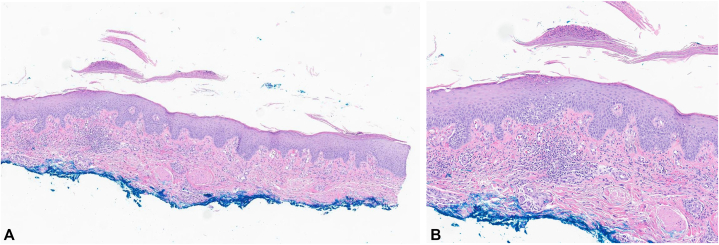
Histology of a skin biopsy of the **(A)** left anterior leg at 4× and **(B)** 20× magnification.

Shave biopsy demonstrated psoriasiform dermatitis with mounded parakeratosis containing neutrophils, loss of the granular layer, and underlying moderate superficial perivascular and interstitial lymphohistiocytic infiltrate with rare neutrophil and extravasated erythrocytes (Fig 2). Upon further questioning, the patient reported a distant history of mild psoriasis, in off treatment remission for 6 years.

Tralokinumab was discontinued, and treatment was initiated with upadacitinib 15 mg orally once daily, in combination with halobetasol propionate-tazarotene 0.01%/0.045% topical cream. After 16 weeks of upadacitinib treatment, clinical examination showed marked improvements with significant clearance of psoriatic lesions (BSA 3%, PGA 1) with continued stability of his AD (Fig 1, *B*).

## Discussion

The current therapeutic approach for AD primarily targets the T-helper 2 (Th2)- mediated immune response, particularly the IL-4 and IL-13 signaling pathways.^4^ In vivo human immunology data reveal that the IL-4/IL-13 axis may act as a negative regulator of the Th17-mediated pathways; therefore, interference with Th2 cytokine signaling may shift immune balance toward Th17 and unmask or amplify previously restrained Th17-driven inflammation, thus potentially contributing to Th-17 mediated disease states such as psoriasis.^4^, ^5^, ^6^ Guttman-Yassky et al further proposed that AD and psoriasis may exist along a Th2-Th17- inflammatory spectrum, particularly within specific AD subtypes, in which patients demonstrate variable immune axis dominance or coexistence.^7^ Accordingly, modulation of one pathway may alter the relative balance of the other cytokine axis.^7^

Dupilumab, an IL-4 and IL-13 receptor antagonist, has been associated with multiple cases of psoriasis and psoriasiform dermatitis. A scoping review on dupilumab-induced psoriasis and psoriasiform dermatitis identified 45 patients with de novo psoriasis and three patients with flares of preexisting psoriasis following dupilumab therapy.^6^ However, only a single clinical case of tralokinumab-induced psoriasis relapse has been reported in the literature. A 48 year old man with a history of dormant childhood psoriasis was treated with tralokinumab for late-onset AD and initially responded well; however, 3 months after tralokinumab initiation, he developed a severe exacerbation of biopsy-proven psoriasis unresponsive to topical therapy.^8^ Methotrexate 12.5 mg weekly induced remission of both AD and psoriasis.^8^

In contrast, our case of tralokinumab-associated psoriasis relapse demonstrated a markedly delayed onset, occurring nearly 2 years after initiation of therapy. The patient responded favorably to oral upadacitinib, a JAK inhibitor approved for the treatment of AD. Upadacitinib modulates multiple inflammatory pathways by inhibiting signaling of cytokines involved in AD, such as IL-4 and IL-13, and psoriatic inflammation, including IL-23.^9^^,^^10^ This therapeutic response highlights the potential of JAK inhibitors for patients with coexisting AD and psoriasis, particularly when biologic therapies are insufficient. The rapid disease relapse following discontinuation of upadacitinib further underscores the importance of ongoing immune modulation in maintaining disease control, highlighting the clinical principle of early, high intensity intervention to achieve disease remission.

This case report contributes to the limited literature on tralokinumab-associated psoriasis and highlights a unique clinical presentation and management approach. These findings suggest that psoriasis risk assessment should be performed before initiating therapy with IL-4 or IL-13 inhibitors, with targeted patient counseling to facilitate early symptom recognition and support shared decision-making. Further, clinicians should remain vigilant for late-onset psoriasis in patients receiving IL-4 or IL-13 targeted therapy, even during prolonged periods of disease stability, particularly in patients with a history of psoriasis. Greater investigation is needed to clarify the mechanism and clinical relevance of this delayed adverse effect and to better elucidate patient-specific risk factors that may guide individualized treatment strategies.

## Conflict of interest

Zarabian’s work is funded through an independent research grant from Galderma. Farah’s work is funded through independent research grants from Incyte and Johnson & Johnson. Zahn is an employee and shareholder of Regeneron Pharmaceuticals. Friedman is a a speaker for Regeneron, Sanofi, Pfizer, Novartis, Arcutis, Johnson & Johnson, Incyte, Galderma, Eli Lilly, and UCB; consultant for La Roche Posay, Galderma, Kenvue, MicroCures, LEO Pharma, Pfizer, Hoth Therapeutics, Zylo Therapeutics, MINO Labs, Johnson & Johnson, Arcutis, Eli Lilly, Novartis, UCB, Regeneron, Sanofi, Takeda, and CeraVe; and received research grants from Pfizer, Eli Lilly, Galderma, Incyte, Johnson & Johnson, and AbbVie.
